# Expansion of the pRAS3 Plasmid Family in *Aeromonas salmonicida* subsp. *salmonicida* and Growing Evidence of Interspecies Connections for These Plasmids

**DOI:** 10.3390/antibiotics11081047

**Published:** 2022-08-03

**Authors:** Kim C. Fournier, Valérie E. Paquet, Sabrina A. Attéré, Judith Farley, Hélène Marquis, Hubert Gantelet, Christian Ravaille, Antony T. Vincent, Steve J. Charette

**Affiliations:** 1 Institut de Biologie Intégrative et des Systèmes (IBIS), Université Laval, Quebec City, QC G1V 0A6, Canada; kim.fournier.3@ulaval.ca (K.C.F.); valerie.paquet@criucpq.ulaval.ca (V.E.P.); sabrina.attere.1@ulaval.ca (S.A.A.); antony.vincent@fsaa.ulaval.ca (A.T.V.); 2 Département de Biochimie, de Microbiologie et de Bio-Informatique, Faculté des Sciences et de Génie, Université Laval, Quebec City, QC G1V 0A6, Canada; 3 Centre de Recherche de l’Institut Universitaire de Cardiologie et de Pneumologie de Québec (IUCPQ), Quebec City, QC G1V 4G5, Canada; 4 Aquarium du Québec, Quebec City, QC G1W 4S3, Canada; farley.judith@sepaq.com; 5 Department of Microbiology and Immunology, College of Veterinary Medicine, Cornell University, Ithaca, NY 14853, USA; hm72@cornell.edu; 6 Ceva Biovac, 49070 Beaucouzé, France; hubert.gantelet@ceva.com; 7 Socsa Elevage, Saint-Charles, 81300 Graulhet, France; c.ravaille@socsa.fr; 8 Département des Sciences Animales, Faculté des Sciences de L’agriculture et de L’alimentation, Université Laval, Quebec City, QC G1V 0A6, Canada

**Keywords:** *Aeromonas salmonicida* subsp. *salmonicida*, antibiotic resistance, insertion sequence, plasmid, pRAS3, tetracycline

## Abstract

*Aeromonas salmonicida* subsp. *salmonicida* is a pathogenic bacterium responsible for furunculosis in salmonids. Following an outbreak of furunculosis, the infection can be treated with antibiotics, but it is common to observe ineffective treatment due to antibiotic resistance. This bacterium has a wide variety of plasmids responsible for this resistance. Among them, pRAS3 carries a tetracycline resistance gene. Several variants of this plasmid have been discovered over the years (pRAS3-3432 and pRAS3.1 to 3.4). During the present study, two new variants of the plasmid pRAS3 were identified (pRAS3.5 and pRAS3-3759) in strains of *A. salmonicida* subsp. *salmonicida*. Plasmid pRAS3-3759, which has been found in many strains from the same region over the past three years, has an additional genetic element identical to one found in pRAS3-3432. This genetic element was also found in *Chlamydia suis*, a swine pathogen. In this study, we analyzed the bacteria’s resistance to tetracycline, the number of copies of the plasmids, and the growth of the strains that carry five of the pRAS3 variants (pRAS3.3 to 3.5, pRAS3-3432, and pRAS3-3759). The results show no particular trend despite the differences between the plasmids, except for the resistance to tetracycline when analyzed in an isogenic background. Blast analysis also revealed the presence of pRAS3 plasmids in other bacterial species, which suggests that this plasmid family has widely spread. This study once again highlights the ability of *A. salmonicida* subsp. *salmonicida* to adapt to furunculosis antibiotic treatments, and the still-growing family of pRAS3 plasmids.

## 1. Introduction

*Aeromonas salmonicida* subsp. *salmonicida* is a Gram-negative bacterium that causes difficulties for the fish farming industry. This opportunistic pathogen is responsible for furunculosis in salmonids, a contagious bacterial disease. It causes high treatment costs and production losses due to mortality [1]. Following an infection, the disease can be treated with antibiotics, but this promotes the appearance of resistant strains, which renders antibiotic treatments ineffective [2]. 

This bacterium is well known for its wide variety of plasmids [3]. Among this plasmidome, some plasmids confer tetracycline monoresistance, such as pRAS3 and pAsa10. In Quebec (Canada), pAsa10, a Tn*1721*-like bearing plasmid, was described once in strain SHY15-2743, which was isolated in 2015. In fact, in this plasmid, the resistance to tetracycline is supported by the *tet* region found on transposon Tn*1721* [4]. 

pRAS3 is a family of IncQ plasmids. To date, five pRAS3 variants have been identified in strains of *A. salmonicida* subsp. *salmonicida*: pRAS3.1, 3.2, 3.3, 3.4, and pRAS3-3432 [5]. The first variants of this family to be completely sequenced were pRAS3.1 and pRAS3.2 [6]. Thus far, these two variants have been found in strains from Europe (Norway and Scotland) and Japan [6,7,8]. Then, pRAS3.3 and pRAS3.4 were identified in strains from North America. pRAS3.3 has been identified several times in different regions of North America, while pRAS3.4 has only been identified once in strain 2010-47 K18 from New Brunswick (Canada) [9,10]. Finally, pRAS3-3432 was discovered in strain SHY16-3432 from Quebec. pRAS3.2 was isolated from *A. salmonicida* sp. in Japan and atypical *A. salmonicida* in Norway, while all the other pRAS3 variants known thus far were isolated from *A. salmonicida* subsp. *salmonicida* [5,8,9].

All these variants contain *tet*(C) (previously described as *tet*(A) [9]) and *tetR* genes, which cause resistance to tetracycline [11]. The *tet*(C) gene mediates tetracycline efflux pumps, so this resistance is energy-dependent. This pump, made of transmembrane proteins, exports tetracycline out of the cell, which reduces the concentration of tetracycline antibiotic in it [12,13]. In the case of pRAS3.4, no complete sequence is available thus far; therefore, its tetracycline resistance gene is still elusive.

The backbone of pRAS3 variants only differs in terms of sequence repeats at two specific sites: Region A (RegA) and Region B (RegB) [9]. RegA is located in the promoter region of the *mobB-mobA/repB* gene, and RegB is located near the *oriV* region. RegA has a varying number of perfect 6 bp repeats, while RegB has a varying number of imperfect 22 bp repeats, depending on the type of pRAS3 plasmid [14]. Loftie-Eaton et al. performed a study on *Escherichia coli* as a surrogate host, which revealed that the number of repetitions at RegA and RegB on pRAS3 (using pRAS3.1, pRAS3.2, and modified versions of these plasmids) could modulate the plasmid copy number (PCN) in the cell [6]. Their study suggested that the presence of an additional repeat at RegA resulted in an increased PCN, while a lower number of repeats at RegB also led to a higher PCN.

Since it bears a unique number of repeats at RegA and RegB, pRAS3-3432 is a particular version of this plasmid. Moreover, pRAS3-3432 has an additional 2 kb insertion sequence (IS) that has also been found in *Chlamydia suis*, a swine pathogen [5]. *C. suis*, an obligate intracellular bacterium, is a pathogen found in the intestinal tract of pigs [15]. Furthermore, the *tetR* gene is altered in pRAS3-3432 due to the insertion sequence, since this IS (IS*cs605*) is localized between a replication-like protein and the *tetR* gene, thus causing a 45-nucleotide deletion at the end of the gene [5]. IS*cs605* is a part of the TetC island found in *C. suis*, a genomic island that confers tetracycline resistance [15]. Massicotte et al. described the high similarity between pRAS3-3432 and the TetC island, therefore suggesting potential horizontal gene transfer between *A. salmonicida* subsp. *salmonicida* and *C. suis* [5].

According to bacteriological analyses carried out at the Faculty of Veterinary Medicine of the University of Montreal on salmonids from fish farms, tetracycline resistance has been on the rise in Quebec for *A. salmonicida* subsp. *salmonicida* since 2017. Over the years, our group has accumulated more than 250 strains of this bacterium. Most of these strains come from Quebec (Canada), while many are from the northeastern part of the United States or from Europe, and a few from the rest of the world. In the present study, we screened various tetracycline-resistant strains of *A. salmonicida* subsp. *salmonicida* to identify the plasmids responsible for this resistance. Plasmid pAsa10 and two new variants of pRAS3 were detected. The sequences of the new pRAS3 variants were characterized, as well as their impact on the physiology of the bacteria bearing them.

## 2. Results

In the present study, we were particularly interested in strains of *A. salmonicida* subsp. *salmonicida* that present monoresistance to tetracycline; this type of resistance has until now been typically associated with the presence of the pRAS3 or pAsa10 plasmids in the bacteria [3]. A PCR-based genotyping was performed on these tetracycline resistant strains to identify the type of plasmid they carry, using the primer pairs presented in Table S1. A total of 18 new strains carrying a pRAS3 plasmid were identified (see strains marked with an asterisk in Table 1). It is interesting to note that in Quebec, strains bearing pRAS3 are on the rise, particularly in one specific region since 2018 (Table 1, region C). Before 2016, no pRAS3 plasmid had been detected in the province of Quebec among the strains studied. The first pRAS3 plasmid detected in this province was the pRAS3-3432 variant [5]. The screening also revealed the presence of pAsa10 in two strains from Quebec (SHY19-3932 and SHY20-1481). 

**Figure 1:**
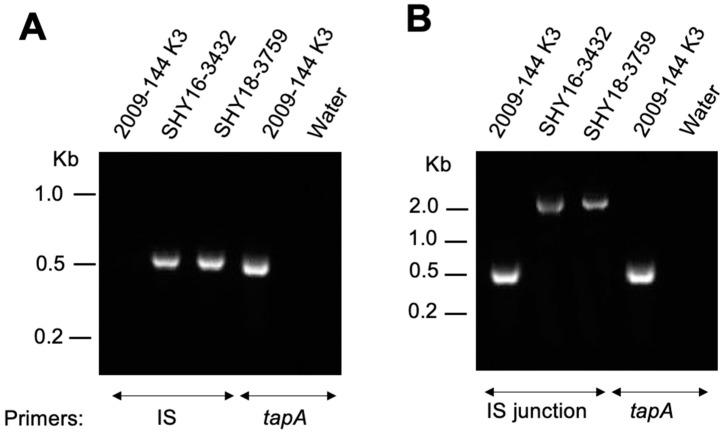
Example of result obtained following screening showing the presence of the IS in strains of *A. salmonicida* subsp. *salmonicida.* pRAS3 in strain 2009-144 K3 does not carry the IS, while the one in SHY16-3432 does [5]. Strain 2009-144 K3 and water were used as positive and negative controls, respectively. (**A**) Genotyping by PCR targeting the IS found in pRAS3-3432. (**B**) Genotyping by PCR targeting the region between the backbone of pRAS3 and the *tetR* gene.

Considering the discovery of pRAS3-3432 in the past, pRAS3 plasmids found over the years have been characterized to verify if some might also have the same IS as pRAS3-3432. PCR genotyping with primers that target the IS revealed that other strains, all from the same region in Quebec (but a different region compared to pRAS3-3432), carry the same IS (Figure 1 and Table 1). In Figure 1A, strains showing an amplicon of about 500 bp have the IS in question, and this IS is at the same position in the plasmid as suggested by positive PCR results with primers that target the IS junction (Figure 1B). 

**Figure 2:**
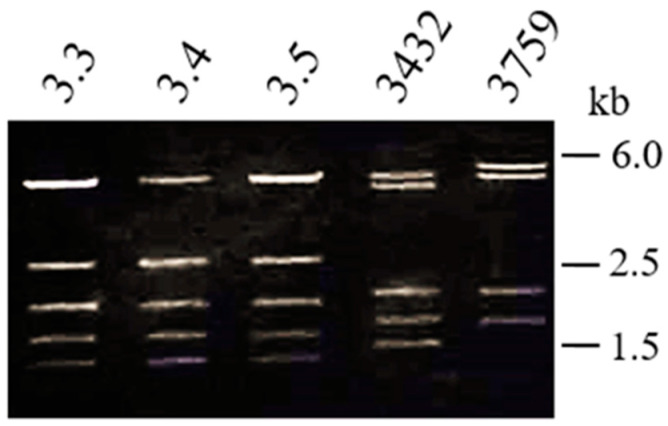
Enzymatic digestion of the pRAS3 plasmids in *E. coli* DH5α. The enzymes *SalI*-HF and *ClaI* were used.

Further analyses were needed to confirm that these pRAS3 plasmids were indeed pRAS3-3432 or a new variant. To compare the sequence of pRAS3.3 and pRAS3-3432, a combination of two restriction enzymes (*SalI* and *ClaI*) was identified to distinguish those plasmids by restriction enzyme digestion profile. However, it is difficult to properly characterize only one plasmid in *A. salmonicida*, since this species carries several other plasmids [3]. Thus, the different plasmids of the pRAS3 family were transferred into *E. coli* DH5α, in order to clearly analyze them without interference by other plasmids. Following enzymatic digestion, one of the tested plasmids showed a different plasmid profile from pRAS3-3432, as shown in Figure 2, even though both variants have the same insertion sequence at the same localization in the plasmid (Figure 1B). Eight strains, all from the same region (Region C, Table 1), were identified as carriers of this new variation of the pRAS3 plasmid, which was named pRAS3-3759 since it was first observed in strain SHY18-3759. 

The screening also revealed that strain 96049, from New York state, also has a pRAS3 that confers resistance to tetracycline. It was therefore also tested during enzymatic digestion, and revealed a plasmid profile identical to pRAS3.3. It seemed relevant to sequence the DNA of this strain, since it is from New York and most of our other strains come from Quebec. Genomic sequencing revealed that this strain has a pRAS3.4 plasmid; this variant has only been identified once before, in a strain from New Brunswick, and has not been completely sequenced in the past [9].

Strain 28516 is the only strain in this study that does not come from North America: it is originally from France. We discovered that this strain has a pRAS3 plasmid, and tested the plasmid during the enzymatic digestion. This plasmid profile did not show anything particular, since it was identical to pRAS3.3. Given its origin, it was still relevant to send it for further genomic sequencing, even if the enzymatic digestion did not show any specific distinction with the already discovered pRAS3 plasmids (Figure 1). The sequencing of the *A. salmonicida* subsp. *salmonicida* 28516 isolate led to the discovery of a new variation of pRAS3, which was named pRAS3.5. 

**Figure 3:**
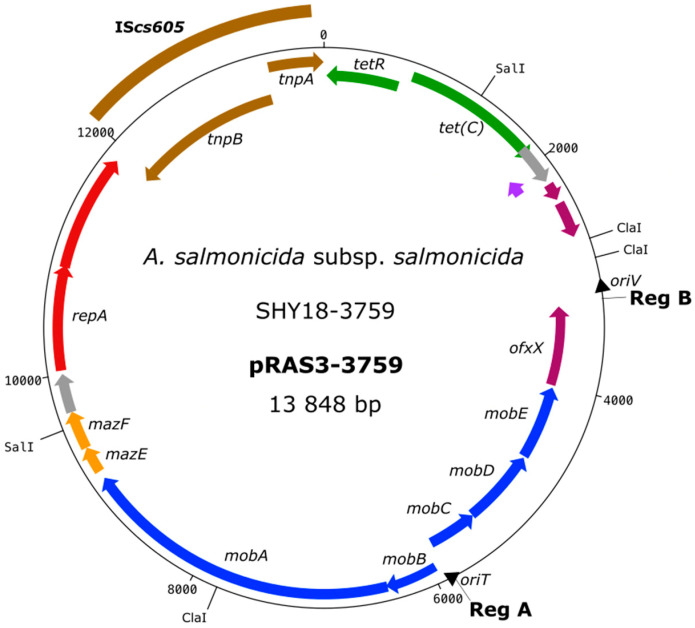
Plasmid map of pRAS3-3759. Genes are represented by arrows. Genes more on the outside are transcribed clockwise, while genes on the inside are transcribed counterclockwise. Green arrows represent genes coding for antibiotic resistance, while the brown arrows represent the genes coding for the transposition of the IS*sc605*. Orange arrows code for an active toxin/antitoxin *pemk-like* system. Recognition sites of the restriction enzymes used for enzymatic digestion (*SalI* and *ClaI*) are shown outside the ring.

Table 2 shows the variations between the different types of pRAS3, which are divergences of the nucleotide repeats at two specific sites of the plasmid, namely, RegA and RegB. Five variants of this plasmid have already been discovered: one variant presenting the IS (pRAS3-3432) and four variations presenting minimal differences [5,9]. The pRAS3.5 plasmid shows four repetitions at RegA and five repetitions at RegB, which is a unique combination of repetitions at these two specific sites. Table 2 shows that pRAS3-3759 has the same number of repeats at RegA and RegB as pRAS3.3. The sequence of pRAS3-3759 has differences from pRAS3-3432, even though the two plasmids have the same IS at the same place in the plasmid. First, the *tetR* gene is altered compared to other pRAS3 plasmids, but not in the same way as pRAS3-3432. In pRAS3-3759, the *tetR* gene has a deletion of 12 nucleotides, compared to pRAS3-3432, which has a deletion of 45 nucleotides in this gene. Furthermore, pRAS3-3759 displays one fewer RegA repeat than pRAS3-3432; in fact, this plasmid is identical to pRAS3.3, but with an additional IS. In pRAS3-3759, the IS encodes a transposase (*tnpA*) and an RNA-guided endonuclease (*tnpB*), like in pRAS3-3432 [5,16]. Like the other variants of the pRAS3 family, pRAS3-3759 also bears two ORFSs (*mazF* and *mazE* genes) that code for a toxin/antitoxin *pemk*-like system (Figure 3) [9].

The *E. coli* strains bearing the available pRAS3 (pRAS3.3, pRAS3.4, pRAS3.5, pRAS3-3432, and pRAS3-3759) were tested for tetracycline MIC to remove the effect of genetic background diversity found in the various *A. salmonicida* subsp. *salmonicida* strains. MICs were determined with a Tecan Infinite 200 PRO microplate reader as performed in the past [5]. pRAS3-3432 has the lowest MIC in this bacterium compared to the other pRAS3 variants tested, while the two new variations, pRAS3-3759 and pRAS3.5, have MICs of 256 µg/mL and 128 µg/mL, respectively (Table 3). 

The number of copies per cell of the different pRAS3 plasmids was determined from the genomic sequences in order to study the impact of the number of repetitions at RegA and RegB on the PCN, as proposed in a previous study [14]. Table 4 presents the results of the number of copies and the number of repeats found at the two specific sites of the plasmid for five variants. There does not appear to be a correlation between the number of repeat sequences and the number of copies of the plasmid per cell, except that the smaller number might be related to the plasmid with the fewest repeats at RegB. However, the study did not establish a clear link between these two parameters.

**Figure 4:**
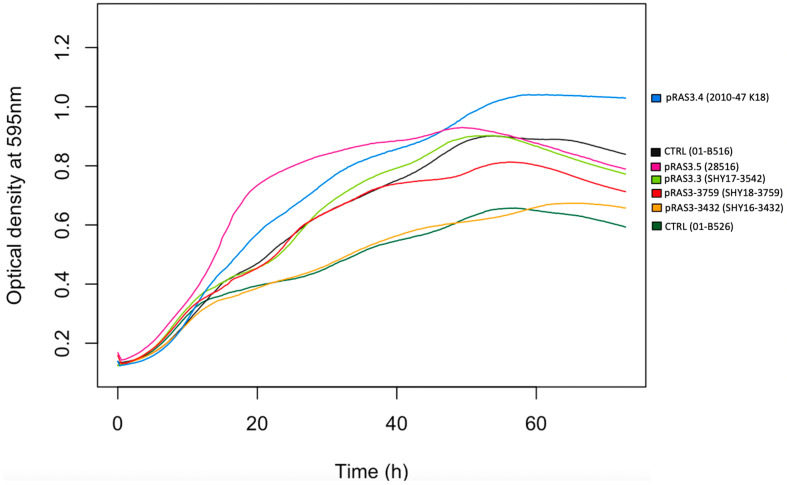
Growth curves at 18 °C of the various pRAS3 plasmids in their strains of *A salmonicida* subsp. *salmonicida*. Two control strains that do not contain pRAS3 were tested. The name of the strains used for the experiment are indicated for each of the curves shown. The strains are shown in Table 1.

**Figure 5:**
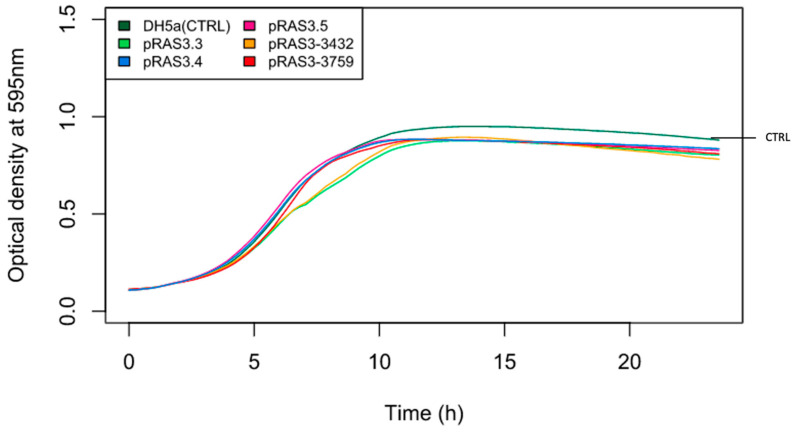
Growth curves at 37 °C of *E. coli* DH5α clones that bear the various pRAS3 variants.

To analyze the impact of the IS present in some pRAS3 variants on the growth of the bacteria, growth curves were performed for strains of *A. salmonicida* subsp. *salmonicida* with different versions of pRAS3 (Figure 4) and for *E. coli* clones (Figure 5). Growth curves with standard deviations are shown in the Supplementary data (Figures S1 and S2). For *A. salmonicida* subsp. *salmonicida*, the strains displayed perceptible growth variation. However, it is not possible to assert that the IS has an impact on the bacterial growth, since it is relatively similar to the growth of the control strains, bearing no plasmids, or other strains bearing the other pRAS3 variants. The strain that has pRAS3.5 grows more quickly in the beginning compared to other strains. Strains that bear pRAS3.4 grow at an OD higher than all the other strains, but its initial exponential growth was similar to one of the other tested strains (Figure 4). Considering the variability between the replicates, the improved growth for pRAS3.4 is not significant (Figure S1). For *E. coli*, there is no visible tendency, except that some strains that have pRAS3 plasmids grow more slowly than the control strain. However, the maximum OD reached by all the clones is quite similar (Figure 5 and Figure S2). 

## 3. Discussion

The present study demonstrates that the pRAS3 plasmid family is very diverse in *A. salmonicida* subsp. *salmonicida*. Seven variants are now known, including five that could be analyzed in parallel in this study for their contribution to the physiology of the bacterium. It also shows that monoresistant plasmids are on the rise in Quebec. Plasmids are a metabolic burden for bacteria, but this does not prevent the increasingly frequent appearance of variations of the pRAS3 plasmid in *A. salmonicida* subsp. *salmonicida*.

Two variants of pRAS3 were identified in this study. pRAS3.5, which comes from France, displays minor differences with pRAS3.1 to 3.4 by bearing a unique number of repetition units at RegA and RegB (Table 2). The new plasmid pRAS3-3759 displays an IS of 2 kb that has only been observed once in pRAS3-3432. This IS is absent from other variants of the pRAS3 family, and was identified in the *tet*(C) island of *C. suis.* In fact, pRAS3-3759 and pRAS3-3432 share a great similarity with the genomic island found in *C. suis* [5]. 

The type of IS found in the pRAS3 plasmids is described as “classic” since these ISs only code for genes essential to their transposition and regulation. The IS*cs605* in pRAS3-3759 codes for a transposase *tnpA* and the *tnpB* gene, which has a role in the regulation of transposition. It has been recently shown that TnpB from *Deinococcus radiodurans* is an RNA-directed nuclease and is proposed to be a precursor to CRISPR-Cas nucleases that can cut DNA [16]. According to the results obtained in our study, the IS does not seem to have an impact on the bacterium, whether at the level of resistance to antibiotics (MIC), the number of copies of the plasmid per cell, or for bacterial growth. 

All strains of *A. salmonicida* bearing pRAS3 plasmids have additional plasmids that may interfere for MIC determination by carrying, for example, genes that can affect the properties of the host cell or other resistance genes that interfere with the resistance selected for the study [17,18]. It is especially the case of strain SHY16-3432, which has another plasmid bearing a tetracycline resistance gene (pAsa5-3432) in addition to pRAS3-3432 [5]. With the goal of testing the pRAS3 plasmids individually without interference, the MICs were determined for tetracycline for *E. coli* DH5α clones that bear pRAS3 variants (except 3.1 and 3.2). The best outcome would have been to test the individual plasmids all in the same *A. salmonicida* subsp. *salmonicida* strain, but the electroporation of pRAS3 plasmids was not possible; multiple attempts were made with no success. 

The results in *E. coli* did not allow us to draw conclusions, since no clear trend was observed, whether the IS was present in the variants or not. pRAS3-3432 has the lowest MIC, but pRAS3-3759 (also with an IS) has an MIC that is four times greater, even if its *tetR* gene, responsible for regulating tetracycline resistance, is also altered due to the presence of the IS. The alteration is a deletion of the C-terminal part of TetR. This part of the protein is involved in the dimerization of the regulator [19]. Perhaps the deletion in TetR protein produced by pRAS3-3432 and pRAS3-3759 has a different impact on the activity of the regulator or leads to inactivation of the regulator in one case and not the other. The lowest MIC was observed for the plasmid with the most important deletion in TetR (pRAS3-3432).

Growth curves were performed on strains with pRAS3 variants, both in *A. salmonicida* subsp. *salmonicida* and in *E. coli.* In *A. salmonicida* subsp. *salmonicida*, the results do not seem to indicate that the various versions of pRAS3 plasmids are harmful for the growth of the bacterium. As shown in Figure 4, we can see that the strains that have pRAS3-3432 or pRAS3-3759 (therefore with IS) grow at a rate within the growth interval of the two control strains, which implies that the strains grow normally. The pRAS3 plasmid is not the only influence in these strains of *A. salmonicida* subsp. *salmonicida*; the other plasmids and other genetic elements present in the bacterium could also affect bacterial growth. These results do not allow any conclusions to be drawn. 

The same observation can be made with *E. coli*. Figure 5 shows the growth curves of the DH5α bacterium. There is no visible trend between clones with the various versions of pRAS3, even though the host bacterium only carries the plasmid pRAS3. Still, it is important to consider that the plasmids have been transferred into another bacterium, since it was not possible to introduce these plasmids into a strain of *A. salmonicida* subsp. *salmonicida* without plasmids. The genetic background of *E. coli* is different from *A. salmonicida* subsp. *salmonicida*, so there could be important influencing elements that may not be active in the recipient bacterium. 

A previous study had linked the number of RegA and RegB repeats to the number of plasmid copies per cell, demonstrating that a higher number of repetitions at RegA would be responsible for a higher PCN, while a lower number of repetitions at RegB would also be responsible for a higher PCN [6]. However, the study only compared two types of pRAS3 at the time, pRAS3.1 and pRAS3.2, and in lab-modified versions of these plasmids in *E. coli*. In our study, we tested that aspect directly in *A. salmonicida* subsp. *salmonicida*. Furthermore, their method of PCN determination was different from ours. They determined this number using qPCR, while we used high-throughput sequencing. The numbers for pRAS3.1 and pRAS3.2 seemed quite high compared to those we obtained; there might be an impact of the method used, but other parameters can influence the results. Our study on five variants of this family (Table 4) suggests that this link between these two parameters may be more complicated than previously estimated. In fact, there seems to be no correlation or tendency between the PCN and the number of repeats at regions A and B of the plasmid. The strain that bears pRAS3.4 has the higher PCN, but there seems to be no impact on the MIC or the bacterial growth. 

The family of pRAS3 plasmids is found in *A. salmonicida* subsp. *salmonicida*, but also in other species. In 2019, when the article describing pRAS3-3432 was published, the version of pRAS3 known at the time with an IS was found only in *A. salmonicida* subsp. *salmonicida* and *C. suis* [5]. Since then, the databases have been enriched and we now find pRAS3 plasmids (with an IS) in other bacterial species, such as *Edwardsiella tarda* and *Klebsiella* sp., which are both pathogenic agents found in farming conditions. *E. tarda* has a variety of hosts, including fish and humans. It is responsible for infections in the fish farming industry, infections which are often associated with poor water quality and warmer temperatures. Furthermore, this bacterium is a risk for zoonoses since it is associated with gastro-intestinal infections in humans [20]. As for *Klebsiella* sp., it is a ubiquitous bacterium and is an opportunistic pathogen for humans and other animals, such as farm animals [21]. Already, in 2019, the link between *A. salmonicida* and *C. suis* created an interesting relation between swine production and aquaculture production for the spread of antibiotic resistance genes, since this was an indicator of horizontal gene transfer (even if the direction of the genetic exchange is unknown). However, the newly discovered species that have pRAS3 plasmids further confirm this link. Furthermore, versions of pRAS3 without the IS are found in other Gram-negative species, such as *Chlamydia trachomatis*, *Chlamydia muridarum*, *Edwardsiella piscida*, *Xanthomonas arboricola*, *Klebsiella* sp., *E. coli*, and *C. suis*. This once again highlights the exchange of genetic information between different species.

The great similarity between the two pRAS3 variants that contain an IS in *A. salmonicida* and the *tet*(C) genomic island in *C. suis* strongly suggests a transfer of genetic material between these two bacteria [5]. Roberts et al. suggested that *C. suis* would be very unlikely to participate in gene exchange by conjugation, since it is an obligate intracellular bacterium, which means that it cannot survive for long outside a cell [15,22]. They therefore suggested that the only way this bacterium could have acquired its tetracycline resistance gene (*tet*(C)) is through the action of a second bacterium carrying the gene, which would have co-infected the same cells as *C. suis* [22]. However, the two bacteria occupy very different environments, with one being a fish pathogen with an ideal growth temperature under 20 °C and the other being a swine pathogen, with ideal growth in warmer conditions [15]. The direction of the genetic exchange is therefore unknown at this time and there is nothing to indicate that *A. salmonicida* would be directly involved in the genetic transfer to *C. suis*. 

Florfenicol is, in Quebec, the most used antibiotic to treat furunculosis in fish, while tetracycline (resistance carried by the pRAS3 plasmids) is not used much [23]. According to Canadian and Quebecois reports on animal production and the use of antibiotics, the tetracycline class is the most widely sold class of antibiotics for animal production in Canada, and the pork sector is the one for which the most antibiotics are sold in Canada [24]. Quebec is the leading pork producer in Canada, and this industry is the second largest agri-food sector in Quebec [25]. The results of passive surveillance of antibiotic resistance in Quebec show that a significant proportion of isolates of many pathogenic bacteria of porcine origin are resistant to tetracyclines. Consequently, in Quebec, resistance to tetracyclines is very frequent for swine pathogens [26]. Aquaculture is vulnerable to the introduction of antibiotic resistance genes. Aquatic environments are at risk of contamination by residual antibiotics from the activities of farms near them. Fish farms are dynamic and complicated environments that are easily influenced by environmental conditions [27]. These environments can therefore become reservoirs of a diversity of bacteria which may be resistant not only to the antibiotics used in fish farming, but also to those used in animal production. Thus, since it is unlikely that *C. suis* would transmit pRAS3 plasmid to *A. salmonicida*, it is possible that other bacteria (probably still not identified) from the swine industry or farming in general may serve as a vector between land and aquatic farming.

The results presented in this study suggest that the IS found in pRAS3 plasmids have no real effect on the physiology of the bacterium. However, we can conclude that there are different versions of pRAS3 and that the appearance of variants is more and more frequent. Based on our results, we conclude that there is nothing that favors one version more than another, for now, compared to what was proposed in the past [6]. pRAS3.3 is the most frequently found version; it is the most distributed version, and the regions of origin are different. Monoresistance has been on the rise in Quebec in recent years, with the identification of pRAS3 in the genome of strains of *A. salmonicida* subsp. *salmonicida* analyzed. However, it is important to keep in mind that the appearance of pRAS3-3759 is particular, since the strains with this version of the plasmid all come from the same fish farming region.

## 4. Materials and Methods

### 4.1. Bacterial Strains and Growth Conditions

The *A. salmonicida* subsp. *salmonicida* strains used in this study are described in Table 1. They were grown from frozen stocks on furunculosis agar (FA) for 3 days at 18 °C as previously described [28]. *E. coli* DH5α was grown from frozen stock on tryptic soy agar (TSA) (Wisent, St-Bruno, QC, Canada) for one day at 37 °C. 

### 4.2. PCR Analyses

DNA lysates of the strains were obtained using a previously described protocol, with PCR mixtures and conditions [18]. The primers required for the present study were designed using the tools available on the Integrated DNA Technologies (IDT) website. The PCR program was as follows: 2 min 30 s at 95 °C, 30 cycles of 30 s at 95 °C, 30 s at 55 °C, and 30 s at 68 °C, followed by a final extension of 10 min at 68 °C. The products were separated on 1.5% agarose gels, which were mixed with 0.5 µg/mL EtBr before casting. Positive and negative controls were also performed and the PCR assay was performed at least twice. All the primers used in this study are listed in Table S1. 

### 4.3. DNA Extraction and Sequencing

The total DNA of strains 28516, 96049, and SHY18-3759 was extracted using DNeasy Blood and Tissue kits (Qiagen, Toronto, ON, Canada) following the manufacturer’s protocol. A step of RNAse treatment at 20 µg/mL was added before proteinase K lysis. Sequencing libraries were prepared from purified bacterial DNA using the Nextera XT DNA Library Preparation Kit, and the sequencing was performed using a MiSeq instrument (Illumina, San Diego, CA, USA) at the Plateforme d’Analyse Génomique of the Institut de Biologie Intégrative et des Systèmes (Université Laval, Quebec City, QC, Canada).

### 4.4. Sequence Assembly and Analyses

The sequencing reads were de novo assembled using A5-miseq version 20160825 [29]. Then, CONTIGuator version 2.7.5 was used for mapping the contigs on the reference genome of strain A449 (NC_009348.1). The unmapped contigs were screened using BLAST [30] to find the *tet*(C) gene. The contig in each dataset bearing the gene sequence was annotated using Prokka [31]. The plasmid sequence was then further characterized using BLAST [30], verified with Artemis version 16.0.0 [32]. Finally, the plasmid maps were created using DNAPlotter version 18.0.0 [33]. The sequences of pRAS3.4, pRAS3.5, and pRAS3-3759, respectively, from strains 96049, 28516, and SHY18-3759, were deposited in DDBJ/ENA/GenBank under the accession numbers ON814114, ON814115, and ON814113, respectively.

### 4.5. Copy Number of pRAS3 Variants in Their Host Strains

The relative sequencing coverage was used in order to be able to estimate the number of plasmids present in the different strains carrying the pRAS3 variants. Initially, the read assembly carried out de novo made it possible to highlight pRAS3 plasmids present in each of the strains. Then, the reads were filtered with fastp version 0.23.2 [34], and mapped with bowtie2 version 2.4.5 [35]. Generated files were converted with SAMtools version 1.15 [36] in order to facilitate visualization using Qualimap v. 2.2.2d [37] to compare the coverage associated with the plasmids to one of the chromosomes in each case.

### 4.6. Plasmid Electroporation

With the goal of introducing the pRAS3 variants available (pRAS3.3, pRAS3.4, pRAS3.5, pRAS3-3432, and pRAS3-3759) in the same bacterial genetic background, the plasmidic DNA of five *A. salmonicida* subsp. *salmonicida* isolates (Table 1) was extracted using the QIAprep^®^ Spin Miniprep Kit (Qiagen, Toronto, ON, Canada) following the manufacturer’s protocol, with the exception that the bacterial colonies on FA plates were suspended directly in 250 µL of buffer P1. The purified plasmidic DNA was then introduced by electroporation into the bacterium *E. coli* DH5α as previously described [38], with the use of tetracycline (50 µg/mL) as a selective agent during growth to select pRAS3-bearing clones. The DNA of the growing clones of *E. coli* DH5α was then purified using the QIAprep Spin Miniprep kit, and PCR assays confirmed the presence of the pRAS3 variants in the recipient host. One clone per plasmid variant was kept for further analyses. An enzymatic digestion was performed on the plasmidic DNA recovered from the various *E. coli* clones. This digestion was executed at 37 °C using the enzymes *SalI*-HF and *ClaI* (NEB, Whitby, ON, Canada), and the resulting digested DNA was separated on 0.7% agarose gel. The gel was submerged in water containing 0.5 µg/mL of EtBr for 30 min to visualize the DNA bands under UV illumination.

### 4.7. Growth Curves

Various *A. salmonicida* subsp. *salmonicida* strains and *E. coli* DH5α clones were inoculated in 10 mL of tryptic soy broth (TSB) (Wisent, St-Bruno, QC, Canada) and were incubated at 18 °C and 37 °C overnight, respectively. The optical density (OD) was adjusted to 0.1 at 595 nm (OD_595_), and the cultures were incubated at 18 °C (for *A. salmonicida* subsp. *salmonicida*) and 37 °C (for *E. coli*) in a Tecan Infinite 200 PRO microplate reader (Tecan, Baldwin Park, CA, USA). Shaking was set to 200 rpm and the ODs were read automatically every 15 min for 72 h (18°C) and 48 h (37 °C). The assays were performed in triplicate.

### 4.8. Assessment of the Minimum Inhibitory Concentration (MIC) of Tetracycline

The tetracycline MIC of various *E. coli* DH5α clones bearing the pRAS3 plasmids were determined in 48-well plates using a formerly described protocol, with concentrations ranging from 0 to 256 µg/mL being tested [9]. The results were read every 15 min in a Tecan Infinite 200 PRO microplate reader (with shaking at 200 rpm). Growth was evaluated throughout the incubation period, and every assay was performed at least in duplicate. 

## Figures and Tables

**Table 1:** Strains used in this study.

Strain	Fish	Origin *^a^*	Tetracycline Resistance Gene-Bearing Plasmid (pRAS3 and pAsa10)	Presence of the pRAS3-3432 Related IS
01-B526 *^b^*	Brook trout	Qc (E)	None	No
01-B516 *^b^*	Brook trout	Qc (F)	None	No
2009-157 K5	Brook trout	NB	pRAS3	No
2010-47 K18	Brook trout	NB	pRAS3	No
2009-195 K29	Brook trout	NB	pRAS3	No
2009-144 K3 *^c^*	Brook trout	NB	pRAS3	No
SHY16-3432 *^c^*	Brook trout	Qc (F)	pRAS3	Yes
91005 *	Atlantic salmon	NY	pRAS3	No
91009 *	Atlantic salmon	NY	pRAS3	No
96049 *^c^* *	Trout	NY	pRAS3	No
900001 *	Rainbow trout	NY	pRAS3	No
890054 *	Brook trout	NY	pRAS3	No
SHY17-3542 *	Brook trout	Qc (A)	pRAS3	No
SHY17-5108 *	NA	Qc (A)	pRAS3	No
SHY-18-2492 *	Brook trout	Qc (C)	pRAS3	Yes
SHY18-3221 *	Brook trout	Qc (A)	pRAS3	No
SHY18-3759 *^c^* *	Brook trout	Qc (C)	pRAS3	Yes
28516 *^c^* *	Trout	France	pRAS3	No
SHY19-3932 **	Brook trout	Qc (G)	pAsa10	No
SHY19-4656 *	Brook trout	Qc (C)	pRAS3	Yes
SHY19-4655 *	Brook trout	Qc (C)	pRAS3	Yes
SHY19-4654 *	Brook trout	Qc (C)	pRAS3	Yes
SHY20-2575 *	Brook trout	Qc (C)	pRAS3	Yes
SHY20-2715 *	Brook trout	Qc (B)	pRAS3	No
SHY20-3274 *	Brook trout	Qc (C)	pRAS3	Yes
SHY20-3753 *	Brook trout	Qc (C)	pRAS3	No
SHY20-1481**	Brook trout	Qc (G)	pAsa10	No

^*a*^: For strains from Quebec, a code was used to identify the corresponding fish farming region. ^*b*^: These two strains are controls used in this study and do not bear pRAS3 variants or pAsa10 compared to all other strains in this table. ^*c*^: These five strains were used for the plasmid electroporation. *: Strains bearing pRAS3 identified in this study. pRAS3 plasmids present in the other strains were described previously [5,9]. **: Strains bearing pAsa10 identified in this study.

**Table 2:** Repeat sequences at regions A (RegA) and B (RegB) in the seven pRAS3 plasmids found in *A. salmonicida* subsp. *salmonicida*.

Plasmid	RegA	RegB
pRAS3.1	CTCCTTCTCGC (CGGGGG) X4 CGGGGTGTGTCT	TGTCA (GGTTACCACCTGCGCCGGGGGT) X3 GGTTACCACCTGCGCCGGGGGCTTGAATT
pRAS3.2	CTCCTTCTCGC (CGGGGG) X3 CGGGGTGTGTCT	TGTCA (GGTTACCACCTGCGCCGGGGGT) X2 GGTTACCACCTGCGCCGGGGGCTTGAATT
pRAS3.3	CTCCTTCTCGC (CGGGGG) X3 CGGGGTGTGTCT	TGTCA (GGTTACCACCTGCGCCGGGGGT) X3 GGTTACCACCTGCGCCGGGGGCTTGAATT
pRAS3.4	CTCCTTCTCGC (CGGGGG) X3 CGGGGTGTGTCT	TGTCA (GGTTACCACCTGCGCCGGGGGT) X4 GGTTACCACCTGCGCCGGGGGCTTGAATT
pRAS3.5 *^a^*	CTCCTTCTCGC (CGGGGG) X4 CGGGGTGTGTCT	TGTCA (GGTTACCACCTGCGCCGGGGGT) X5 GGTTACCACCTGCGCCGGGGGCTTGAATT
pRAS3-3432	CTCCTTCTCGC (CGGGGG) X2 GCGGGGTGTGTCT *^b^*	TGTCA (GGTTACCACCTGCGCCGGGGGT) X6 GGTTACCACCTGCGCCGGGGGCTTGAATT
pRAS3-3759 *^a^*	CTCCTTCTCGC (CGGGGG) X3 CGGGGTGTGTCT	TGTCA (GGTTACCACCTGCGCCGGGGGT) X3 GGTTACCACCTGCGCCGGGGGCTTGAATT

***^a^***: pRAS3 variants identified in this study. ***^b^***: pRAS3-3432 has an additional nucleotide at RegA (in red), making it the only variant without a perfect repetition at this site.

**Table 3:** Tetracycline resistance induced by pRAS3 plasmids in *E. coli*.

Plasmid	MIC (µg/mL) for Tetracycline *^a^*
pRAS3.3	128
pRAS3.4	256
pRAS3.5	128
pRAS3-3432	64
pRAS3-3759	256

*^a^* Growth was assessed after 24 h.

**Table 4:** Plasmid copy number (PCN) per cell for different pRAS3 plasmids.

Isolate	Type of pRAS3 *^a^*	Copy number of pRAS3 *^b^*	Number of Repeats at RegA	Number of Repeats at RegB
SHY16-3432	pRAS3-3432	6	2	6
SHY18-3759 SHY17-3542	pRAS3-3759 pRAS3.3	3 4	3 3	3 3
96049	pRAS3.4	10	3	4
28516	pRAS3.5	4	4	5

***^a^***: pRAS3.1 and pRAS3.2 are not shown in this table because their PCN was determined in the past by Loftie-Eaton et al. (2009) by a different method than that used in this study. Therefore, it was not possible to compare these data with those obtained in this study. ***^b^***: The PCN was determined by considering the number of chromosomes in the cell equal to 1.

## Data Availability

The sequences of pRAS3.4, pRAS3.5, and pRAS3-3759 were deposited in DDBJ/ENA/GenBank under the accession numbers ON814114, ON814115, and ON814113, respectively.

## Supplementary Materials

The following supporting information can be downloaded at: https://www.mdpi.com/article/10.3390/antibiotics11081047/s1, Table S1. Primer pairs used in this study; Figure S1: Growth curves at 18 °C of various *A. salmonicida* subsp. *salmonicida* both with and without the pRAS3 plasmid variants, with standard deviations; Figure S2. Growth curves at 37 °C of *E. coli* DH5α clones with pRAS3 variants, with standard deviations. Refs [9,39,40] are cited in Supplementary Materials.

## References

[1] Dallaire-Dufresne S., Tanaka K.H., Trudel M.V., Lafaille A., Charette S.J. (2014). Virulence, genomic features, and plasticity of *Aeromonas salmonicida* subsp. *salmonicida*, the causative agent of fish furunculosis. Vet. Microbiol..

[2] Charette S.J. (2021). Microbe profile: *Aeromonas salmonicida*: An opportunistic pathogen with multiple personalities. Microbiology.

[3] Vincent A.T., Hosseini N., Charette S.J. (2021). The *Aeromonas salmonicida* plasmidome: A model of modular evolution and genetic diversity. Ann. N. Y. Acad. Sci..

[4] Attéré S.A., Vincent A.T., Paccaud M., Frenette M., Charette S.J. (2017). The Role for the Small Cryptic Plasmids As Moldable Vectors for Genetic Innovation in *Aeromonas salmonicida* subsp. *salmonicida*. Front. Genet..

[5] Massicotte M.-A., Vincent A.T., Schneider A., Paquet V.E., Frenette M., Charette S.J. (2019). One *Aeromonas salmonicida* subsp. *salmonicida* isolate with a pAsa5 variant bearing antibiotic resistance and a pRAS3 variant making a link with a swine pathogen. Sci. Total Environ..

[6] Loftie-Eaton W., Rawlings D.E. (2010). Evolutionary competitiveness of two natural variants of the IncQ-like plasmids, pRAS3.1 and pRAS3.2. J. Bacteriol..

[7] Aoki T., Takahashi A. (1986). Tetracycline-Resistant Gene of a Non-Transferable R Plasmid from Fish-Pathogenic Bacteria *Aeromonas salmonicida*. Nippon Suisan Gakkaishi.

[8] L’Abée-Lund T.M., Sørum H. (2002). A global non-conjugative Tet C plasmid, pRAS3, from *Aeromonas salmonicida*. Plasmid.

[9] Vincent A.T., Trudel M.V., Paquet V.E., Boyle B., Tanaka K.H., Dallaire-Dufresne S., Daher R.K., Frenette M., Derome N., Charette S.J. (2014). Detection of variants of the pRAS3, pAB5S9, and pSN254 plasmids in *Aeromonas salmonicida* subsp. *salmonicida*: Multidrug resistance, interspecies exchanges, and plasmid reshaping. Antimicrob. Agents Chemother..

[10] Gauthier J., Marquis H., Paquet V.E., Charette S.J., Levesque R.C., Derome N. (2021). Genomic Perspectives on *Aeromonas salmonicida* subsp. *salmonicida* Strain 890054 as a Model System for Pathogenicity Studies and Mitigation of Fish Infections. Front. Mar. Sci..

[11] Bertram R., Neumann B., Schuster C.F. (2021). Status quo of tet regulation in bacteria. Microb. Biotechnol..

[12] Chopra I., Roberts M. (2001). Tetracycline antibiotics: Mode of action, applications, molecular biology, and epidemiology of bacterial resistance. Microbiol. Mol. Biol. Rev..

[13] Mascaretti O.A. (2003). Bacteria Versus Antibacterial Agents: An Integrated Approach.

[14] Loftie-Eaton W., Rawlings D.E. (2009). Comparative biology of two natural variants of the IncQ-2 family plasmids, pRAS3.1 and pRAS3.2. J. Bacteriol..

[15] Dugan J., Rockey D.D., Jones L., Andersen A.A. (2004). Tetracycline Resistance in *Chlamydia suis* Mediated by Genomic Islands Inserted into the Chlamydial *inv*-Like Gene. Antimicrob. Agents Chemother..

[16] Karvelis T., Druteika G., Bigelyte G., Budre K., Zedaveinyte R., Silanskas A., Kazlauskas D., Venclovas Č., Siksnys V. (2021). Transposon-associated TnpB is a programmable RNA-guided DNA endonuclease. Nature.

[17] Darphorn T.S., Bel K., Koenders-van Sint Anneland B.B., Brul S., Ter Kuile B.H. (2021). Antibiotic resistance plasmid composition and architecture in *Escherichia coli* isolates from meat. Sci. Rep..

[18] Attéré S.A., Vincent A.T., Trudel M.V., Chanut R., Charette S.J. (2015). Diversity and Homogeneity among Small Plasmids of *Aeromonas salmonicida* subsp. *salmonicida* Linked with Geographical Origin. Front. Microbiol..

[19] Yamasaki S., Nikaido E., Nakashima R., Sakurai K., Fujiwara D., Fujii I., Nishino K. (2013). The crystal structure of multidrug-resistance regulator RamR with multiple drugs. Nat. Commun..

[20] Hassan H.A., Ding X., Zhang X., Zhu G. (2020). Fish borne *Edwardsiella tarda* eha involved in the bacterial biofilm formation, hemolytic activity, adhesion capability and pathogenicity. Arch. Microbiol..

[21] Bagley S.T. (1985). Habitat association of *Klebsiella* species. Infect. Control.

[22] Roberts M.C. (2005). Update on acquired tetracycline resistance genes. FEMS Microbiol. Lett..

[23] Lafaille A. (2014). Rapport des Activités en Ichtyopathologie du 1er Janvier 2013 au 31 Décembre 2013.

[24] Public Health Agency of Canada (2021). Canadian Antimicrobial Resistance Surveillance System Report.

[25] Gouvernement du Québec Élevage Porcin (porc). https://www.quebec.ca/agriculture-environnement-et-ressources-naturelles/agriculture/industrie-agricole-au-quebec/productions-agricoles/elevage-porcin-porc.

[26] Programme Québécois D’antibiosurveillance Vétérinaire Résultat de la Surveillance Passive de L’antibiorésistance. https://www.mapaq.gouv.qc.ca/SiteCollectionDocuments/Santeanimale/Antibioresistance/Rapportannuel2020-Surveillance_passive_antibioresistance.pdf.

[27] Zhao Y., Yang Q.E., Zhou X., Wang F.-H., Muurinen J., Virta M.P., Brandt K.K., Zhu Y.-G. (2021). Antibiotic resistome in the livestock and aquaculture industries: Status and solutions. Crit. Rev. Environ. Sci. Technol..

[28] Daher R.K., Filion G., Tan S.G., Dallaire-Dufresne S., Paquet V.E., Charette S.J. (2011). Alteration of virulence factors and rearrangement of pAsa5 plasmid caused by the growth of *Aeromonas salmonicida* in stressful conditions. Vet. Microbiol..

[29] Coil D., Jospin G., Darling A.E. (2015). A5-miseq: An updated pipeline to assemble microbial genomes from Illumina MiSeq data. Bioinformatics.

[30] Altschul S.F., Gish W., Miller W., Myers E.W., Lipman D.J. (1990). Basic local alignment search tool. J. Mol. Biol..

[31] Seemann T. (2014). Prokka: Rapid prokaryotic genome annotation. Bioinformatics.

[32] Carver T., Harris S.R., Berriman M., Parkhill J., McQuillan J.A. (2012). Artemis: An integrated platform for visualization and analysis of high-throughput sequence-based experimental data. Bioinformatics.

[33] Carver T., Thomson N., Bleasby A., Berriman M., Parkhill J. (2009). DNAPlotter: Circular and linear interactive genome visualization. Bioinformatics.

[34] Chen S., Zhou Y., Chen Y., Gu J. (2018). fastp: An ultra-fast all-in-one FASTQ preprocessor. Bioinformatics.

[35] Langmead B., Salzberg S.L. (2012). Fast gapped-read alignment with Bowtie 2. Nat. Methods.

[36] Li H., Handsaker B., Wysoker A., Fennell T., Ruan J., Homer N., Marth G., Abecasis G., Durbin R., Genome Project Data Processing Subgroup (2009). The Sequence Alignment/Map format and SAMtools. Bioinformatics.

[37] García-Alcalde F., Okonechnikov K., Carbonell J., Cruz L.M., Götz S., Tarazona S., Dopazo J., Meyer T.F., Conesa A. (2012). Qualimap: Evaluating next-generation sequencing alignment data. Bioinformatics.

[38] Dower W.J., Miller J.F., Ragsdale C.W. (1988). High efficiency transformation of *E. coli* by high voltage electroporation. Nucleic Acids Res..

[39] Ebanks R.O., Knickle L.C., Goguen M., Boyd J.M., Pinto D.M., Reith M., Ross N.W. (2006). Expression of and secretion through the *Aeromonas salmonicida* type III secretion system. Microbiology.

[40] Trudel M.V., Tanaka K.H., Filion G., Daher R.K., Frenette M., Charette S.J. (2013). Insertion sequence AS5 (ISAs5) is involved in the genomic plasticity of *Aeromonas salmonicida*. Mob. Genet. Elem..

