# Supplementary material for: Expansion of the pRAS3 Plasmid Family in Aeromonas salmonicida subsp. salmonicida and Growing Evidence of Interspecies Connections for These Plasmids
Source: Antibiotics (Basel). 2022 Aug 3;11(8):1047. doi: 10.3390/antibiotics11081047 (PMC9405359; doi:10.3390/antibiotics11081047)
Supplement: Supplementary file 1 [file antibiotics-11-01047-s001.zip › antibiotics-1817938-SI.pdf]

Supplementary information

Expansion of the pRAS3 Plasmid Family in *Aeromonas salmonicida* subsp. *salmonicida* and Growing Evidence of Interspecies Connections for These Plasmids

Kim C. Fournier[1,2], Valérie E. Paquet[1,2,3], Sabrina A. Attéré[1,2], Judith Farley[4], Hélène Marquis[5], Hubert Gantelet[6], Christian Ravaille[7], Antony T. Vincent[1,8] and Steve J. Charette[1,2,3]*

1. Institut de Biologie Intégrative et des Systèmes (IBIS), Université Laval, Quebec City, QC, Canada, G1V 0A6; kim.fournier.3@ulaval.ca, valerie.paquet@criucpq.ulaval.ca, sabrina.attere.1@ulaval.ca, antony.vincent@fsaa.ulaval.ca, steve.charette@bcm.ulaval.ca
2. Département de biochimie, de microbiologie et de bio-informatique, Faculté des sciences et de génie, Université Laval, Quebec City, QC, Canada, G1V 0A6
3. Centre de recherche de l'Institut universitaire de cardiologie et de pneumologie de Québec (IUCPQ), Quebec City, QC, Canada, G1V 4G5
4. Aquarium du Québec, Quebec City, QC, Canada, G1W 4S3; farley.judith@sepaq.com
5. Department of Microbiology and Immunology, College of Veterinary Medicine, Cornell University, Ithaca, NY 14853, United States; hm72@cornell.edu
6. Ceva Biovac, 49070 Beaucouzé, France; hubert.gantelet@ceva.com
7. Socsa Elevage, Saint-Charles, 81300 Graulhet, France; c.ravaille@socsa.fr
8. Département des sciences animales, Faculté des sciences de l'agriculture et de l'alimentation, Université Laval, Quebec City, QC, Canada G1V 0A6

* Correspondence: steve.charette@bcm.ulaval.ca; Tel.: 1-418-656-2131, ext. 406914

Table S1. Primer pairs used in this study.

Target	Sequence (5'-3') Fw/Rev	PCR product (bp)	Reference
**TapA**	ACATGAAGAAGCAATCAGGC<br>AGAGGTCATGCGTTAGCAG	443	[1]
**pRAS3**	CATGAGCATTGCGGTAGCACTCAA<br>TCGCTTGCGGGAACTTCTCATACT	279	[2]
**RegA**	CGTTGTGGATGTGCTTCAGCAATC<br>GAGTGGTTGAGCAATACAGGATGC	Variable[a]	[3]
**RegB**	GGATAGTCGATCTGCTGGATACG<br>CTACCCTGTGGAACACCTACATCT	Variable[a]	[3]
**IS pRAS3-3432**	TGGTGTGCTCTTCCAGATATTC<br>CCGAACCTATGCCCGTAAAT	494	This study
**IS junction pRAS3-3432**	GGTCAACTACGCGATGCTTTA<br>TGGCAATCAGCTACTTCACC	2456	This study
**pAsa10**	GGCGATAAACGCTTGGCGAAAAG<br>CATCCTGGACTGGCTGCAAAGTG	498	This study

[a]: Depending on the number of repetitions found in this region, the PCR product may vary from about 400 to 900 bp.

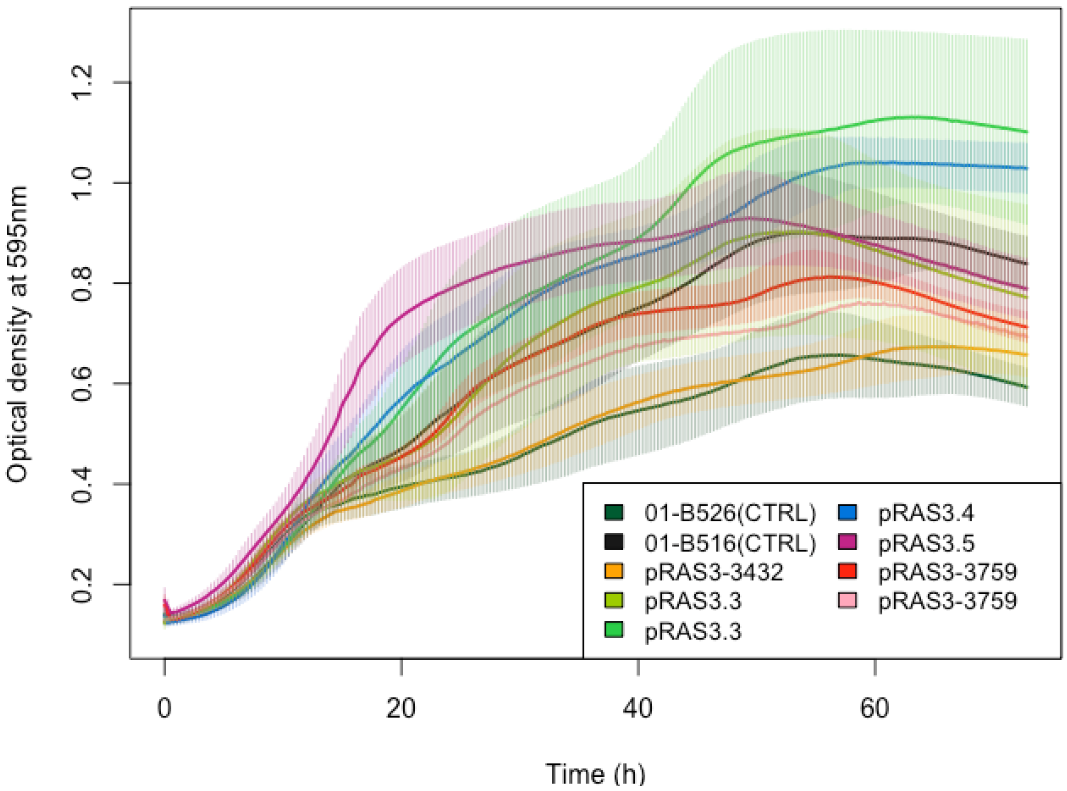

Figure S1: Growth curves at 18 °C of various *A. salmonicida* subsp. *salmonicida* both with and without the pRAS3 plasmid variants with standard deviations.

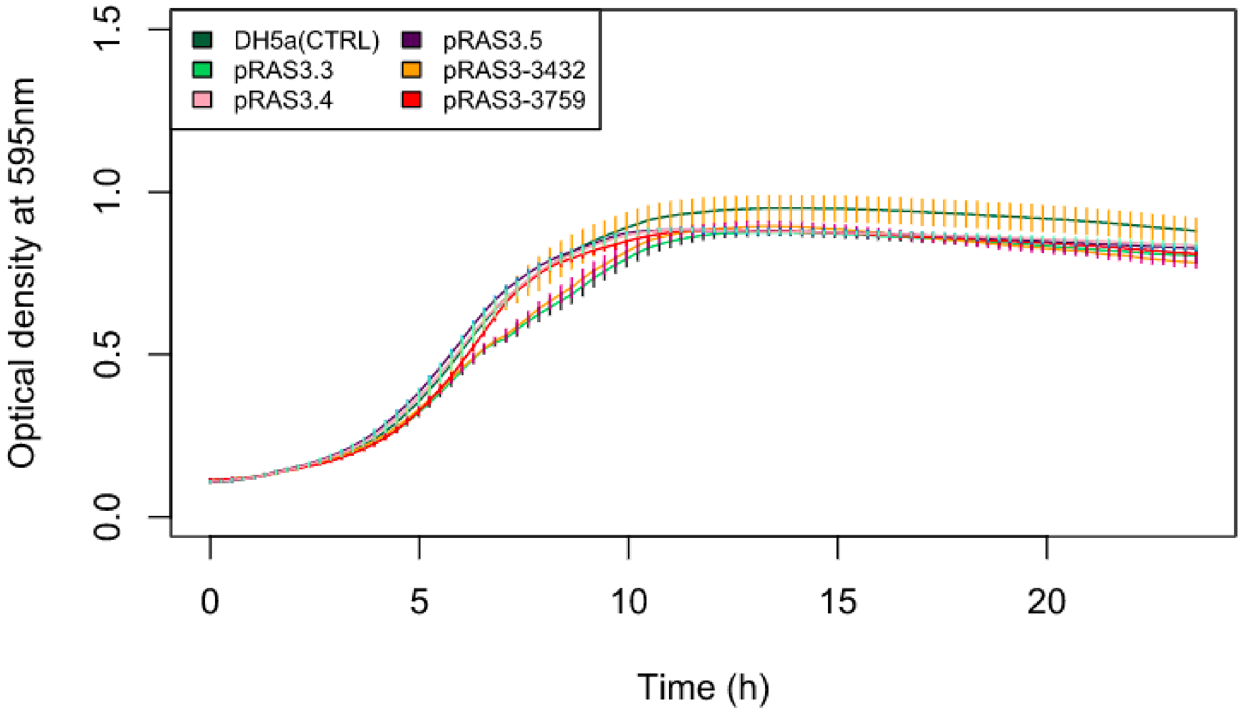

Figure S2. Growth curves at 37 °C of *E. coli* DH5α clones with pRAS3 variants with standard deviations.

References

1. Ebanks, R.O.; Knickle, L.C.; Goguen, M.; Boyd, J.M.; Pinto, D.M.; Reith, M.; Ross, N.W. Expression of and secretion through the *Aeromonas salmonicida* type III secretion system. *Microbiology (Reading)* **2006**, *152*, 1275-1286, doi:10.1099/mic.0.28485-0.

2 Trudel, M.V.; Tanaka, K.H.; Filion, G.; Daher, R.K.; Frenette, M.; Charette, S.J. Insertion sequence AS5 (ISAs5) is involved in the genomic plasticity of *Aeromonas salmonicida*. *Mob Genet Elements* **2013**, *3*, e25640, doi:10.4161/mge.25640.

3. Vincent, A.T.; Trudel, M.V.; Paquet, V.E.; Boyle, B.; Tanaka, K.H.; Dallaire-Dufresne, S.; Daher, R.K.; Frenette, M.; Derome, N.; Charette, S.J. Detection of variants of the pRAS3, pAB5S9, and pSN254 plasmids in *Aeromonas salmonicida* subsp. *salmonicida*: multidrug resistance, interspecies exchanges, and plasmid reshaping. *Antimicrob Agents Chemother* **2014**, *58*, 7367-7374, doi:10.1128/aac.03730-14.
